# Towards cultural materialism in the medical humanities: the case of blood rejuvenation

**DOI:** 10.1136/medhum-2017-011209

**Published:** 2017-05-11

**Authors:** Catherine Oakley

**Keywords:** Popular media

## Abstract

This paper argues for an approach within the medical humanities that draws on the theoretical legacy of cultural materialism as a framework for reading cultural practices and their relationship to the social and economic order. It revisits the origins and development of cultural materialism in cultural studies and literary studies between the 1970s and 1990s and considers how, with adaptation, this methodology might facilitate ideological criticism focused on material formations of health, disease and the human body. I outline three key characteristics of a medicocultural materialist approach along these lines: (a) interdisciplinary work on a broad range of medical and cultural sources, including those drawn from ‘popular’ forms of culture; (b) the combination of historicist analysis with scrutiny of present-day contexts; (c) analyses that engage with political economy perspectives and/or the work of medical sociology in this area. The subsequent sections of the paper employ a medicocultural materialist approach to examine conjectural understandings of, and empirical investigations into, the capacity of transfused human blood to rejuvenate the ageing body. I trace textual faultlines that expose the structures of power which inform the movement of blood between bodies in ‘medical gothic’ fictions from the 19th-century fin de siècle, including Mary Elizabeth Braddon's ‘Good Lady Ducayne’ (1896) and Bram Stoker's *Dracula* (1897). I conclude with a critique of biomedical innovations in blood rejuvenation in the era of medical neoliberalism, before considering the potential applications of medicocultural materialism to other topics within the field of the medical humanities.

## Introduction

Evolving understandings of the human body have reserved a special place for blood since the very earliest attempts to explain life. Its pre-anatomical significance lay in its conception as a hot, moist fluid, akin to a vital force. Subsequent scientific investigations have elucidated the physiological functions of the substance: the delivery of antibodies, nutrients and oxygen to all cells in the body and the transportation of metabolic waste products from those cells. At the same time, the resonant symbolic meanings of blood have been powerfully shaped in the contexts of religious belief and iconography, where it has come to connote sacrifice and renewal, and in relation to race, where it has functioned as ‘the symbolic means to perpetuate, legitimate and corroborate earthly power’ (p. 14).^1^

Existing scholarship has sketched a linear narrative of progress from humoral, mythological and mystical conceptions of blood, to a ‘modern’, mechanistic understanding of the substance. These accounts suggest the displacement of conjectural understanding by empirical knowledge and the transformative, paradigm-altering effects of scientific discovery. They also share two further characteristics: an emphasis on the 19th-century fin de siècle as the historical period in which this change intensified, and a focus on the instrumentality of blood transfusions (in both theory and practice) in accelerating this epistemological shift. Thus, in the first section of his book *Blood: An Epic History of Medicine and Commerce*, the science journalist and author Douglas Starr covers the period from antiquity to the early 1900s as ‘the time when the concept of blood moved from the magical to the biological; when blood became recognised as a therapeutic liquid transfusable from one creature to another’ (p. xiii).^2^ Kim Pelis, writing from the perspective of a medical historian, tells a similar story, noting that the development of scientific instruments ‘for measuring and recording the blood pressure and volume’, representative of new efforts to ‘quantify the circulatory system’, contributed to the formation of a ‘devitalised blood concept’ that was evident in medical knowledge by the early 1880s (p. 194).^3^ More recently, the literary scholar Aspasia Stephanou has focused attention on representations of transfusions in vampire texts published in the last two decades of the 19th century, describing a shift from ‘mythical or symbolic’ interpretations to ‘a more rational understanding of blood as a neutral fluid’ (p. 54).^4^

In this article, I seek to disrupt the principal narrative that holds science to be the dominant discursive authority in our evolving understanding of the meanings and value of blood. I contend that scholarly accounts charting the epistemological triumph of science in relation to blood have deemphasised the enduring power and dynamism of its symbolic and cultural meanings, and overlooked their constitutive role in its ideological formations. In place of a generalised, atheoretical, teleological reading of blood from antiquity to the present day, I offer historicist, materialist readings that are alert to the nexus of social, scientific, economic and cultural factors that have informed its shifting meanings. To do so, I focus on one aspect of blood that has not been extensively critically explored to date—that of blood rejuvenation. In medical encounters and in cultural symbolism, blood has long been a visible index of corporeal damage and death (wounds, haemorrhage, infection). Yet, it has also signified renewal and new life (menstruation, immunology, coagulation). Today, the biology of the blood remains complex, but an emerging line of scientific enquiry is exploring its regenerative character, based on the theory that it may have potential to rejuvenate the ageing body. Although often used in the generalised sense of the term (‘to give new life to; to refresh, reinvigorate’), the verb ‘rejuvenate’ also has the more precise meaning ‘to make young or fresh again; to restore to youth or to the appearance of youth’.^5^ As idea and practice, then, ‘rejuvenation’ corresponds closely with both ‘regeneration’ and ‘longevity’, but also carries a distinctive meaning. While ‘regeneration’ denotes ‘the action of coming or bringing into renewed existence; rebirth; restoration’,^6^ and ‘longevity’ indicates the extension of the lifespan into a future period, ‘rejuvenation’ suggests an idiosyncratic process: reversing the biological clock to recover a former state of youthfulness (‘re’+‘juvenescence’).

Associations between blood and rejuvenation in Western culture have recurred consistently across different time periods, and these textual traces are manifest in a wide range of sources. I focus here on two historical periods in which discussions and representations of blood's capacity to restore the ageing organism to the physiological condition of youth have been distinctly visible: the late 19th-century fin de siècle, and the present day. Blood's rejuvenatory potential was a central preoccupation of both scientists and literary writers in the closing years of the 19th century. Now, over a century later, current scientific research projects investigating cell and tissue ageing and the development of age-related diseases are employing blood-related techniques such as plasma infusions and parabiosis (an experimental procedure in which two living animals of different ages are joined surgically and develop a single, shared circulatory system). Analysing each of these historical moments in turn, and drawing comparative perspectives from their juxtaposition, I situate blood rejuvenation within enduring structures of human control and mastery and assess the continuing implications of this paradigm.

## Cultural materialism and the medical humanities

The emergence of the critical medical humanities has foregrounded the value of ‘experimentation, reflective practice, collaboration and modes of sceptically risky thinking’ within the medical humanities (p. 4).^7^ It insists that scientific knowledge is constituted across a wide range of sites beyond the laboratory and the clinical encounter, and calls for analyses that encompass ‘the many valances of the word “critical”—urgent, sceptical, evaluative and mobilising the long philosophical and political traditions of critique’ (p. 2). This recognition that scientific knowledge does not exist independently of other forms of knowledge but is culturally produced and mediated has strong affinities with a cultural studies approach to the medical humanities. For cultural studies scholars, ‘the entire range of a society's arts, beliefs, institutions and communicative practices’ can only be fully understood in the context of the social, historical and economic structures that informed their production, and vice versa (p. 4).^8^ Bradley E. Lewis heralded ‘the new genre of cultural studies of medicine’ in the late 1990s, in an essay that sought to unify and catalyse such practice:

As of now, cultural studies of medicine is new enough that most of its practitioners are dispersed with minimal sense of connection. The medical community seems to have little awareness of their work, and the cultural studies community, in its more predominant strands, is concerned primarily with popular culture. There is no single place that a reader in this domain can turn for a reliable collection of this work. None of it is available in the medical book store, and there is no dependable “keyword” available for general library searches (p. 11).^9^

Almost 20 years later, this assessment is still largely valid. The challenge here lies in part in the inherent resistance of cultural studies to definition and demarcation, given its heterogeneous methodologies and applications across multiple disciplines. This generic amorphousness need not, however, preclude persistent attempts to agglomerate and mobilise diverse practices across cultural studies of medicine. Indeed, a coming-together of scholars attentive to the social and cultural meanings of medicine as expressed in multiple and diverse cultural forms has the potential to strengthen critique of biomedical innovation and practice within both historical contexts and the current political economy.

One means of achieving these ends, I suggest here, might begin with the reappraisal of cultural materialism, a theoretical movement within cultural studies derived from the Western tradition of Marxist criticism and elaborated initially within the work of the Welsh academic Raymond Williams.^i^ Cultural materialism offers a framework for reading cultural practices and their relationship to the social and economic order that acknowledges structures of power. Williams' seminal contribution to Marxist criticism was to revise the classical conception of culture as a passive, secondary ‘superstructural’ response to, or ‘reflection’ of, a primary economic ‘base’, and to insist on the active agency of cultural ideas, beliefs and practices within material social life (p. 19).^11^ Williams problematised the ‘received category of literature’ as the work of an educational elite, and advocated a critical practice inclusive of diverse cultural forms beyond the literary (pp. 53, 48). From this understanding, the practice of cultural materialism engages in acts of recovery, by revisiting canonical texts and reinscribing forms of writing that are marginal, popular and non-canonical. These texts are conceptualised as ‘productive forces’—a term which, in traditional Marxist analysis, had referred primarily to industry, but which Williams modified to encompass ‘the realm of art and ideas’ commonly referred to as ‘culture’ (pp. 92–94). Williams approached the interplay of ‘productive forces’ as a nexus of ‘dominant’, ‘residual’ and ‘emergent’ elements. The ‘dominant’ (or hegemonic) order, he observed, always faces ‘oppositional’ ideas and practices. The ‘residual’, by definition, has been effectively formed in the past, but is still active in the cultural process (p. 122). The ‘emergent’ refers to ‘new meanings and values, new practices, new relationships and kinds of relationship [that] are continually being created’ (p. 123). Residual and emergent elements can be co-opted by, or subsumed within within, the dominant culture. But they can also work against it. The cultural materialist scholar approaches sources with an eye to these ‘alternative political and cultural emphases’, decoding the ways in which these works and ideas are ‘clearly affected by hegemonic limits and pressures’, but also offer ‘significant breaks beyond them’ (p. 114).

Williams' approach, developed gradually across the late 1970s and early 1980s, was taken up in the mid-1980s and early 1990s by the literary scholars Jonathan Dollimore and Alan Sinfield, who developed cultural materialism as a mode of analysis in their work on early modern literature. In the introduction to their coauthored volume *Political Shakespeare: Essays in Cultural Materialism,* Dollimore identified the key characteristics of this practice: historical contextualisation; close analysis of the ideological force of literary texts at the level of form (language, narrative conventions, style, register, tropes) and a political commitment within literary studies which—as Williams' had been—was rooted in social democratic values (pp. vi–viii).^12^ Elsewhere, Sinfield demonstrated a critical archaeology of ideological ‘faultlines’ in cultural texts.^13^ These he conceptualised as sites of contestation between hegemonic and counterhegemonic concepts of class, gender, sexuality and race—the dynamic junctures of dominant, residual and emergent elements in the social order.

Despite the potential offered by cultural materialism's combination of rigorous historical critique and analysis of contemporary issues and debates, little has been published on the methodology since Dollimore and Sinfield.^ii^ Although elements of its practice correspond with the work of some contemporary scholars within the medical humanities, we might ask why cultural materialism (and indeed, British cultural studies in general), has been slow to converge more fully with the field. The answer may lie, in part, in the fact that the original cultural materialists did not engage substantively with science but focused instead on other structures of power (state, church, academy, language, law, religion, art). An additional contributory factor here may be that literary approaches within the medical humanities have focused predominantly on canonical texts—an adherence that could be interpreted as a natural consequence of their efforts to obtain legitimacy within a shifting and contested critical field. We might also detect a general anxiety within the medical humanities surrounding the notion of medicine-as-institution, and the diagnosis, treatment and prevention of disease and human suffering as sometime instruments of dominant ideologies. As Sara Wasson has observed, cultural studies of medicine that are attentive to ‘the dangers inherent within institutional discourses, marginalisation of disempowered demographics and the often corrosive effects of capitalism on medical practice’ are vulnerable to misreadings and accusations of hostility. Although the cultural medical historian's interest may lie, at least in part, in ‘the shadow side of medicine’, Wasson notes, ‘the presence of these shadows does not deny that medicine is also a thing of light’ (p. 3).^16^

In arguing here for cultural materialism within the medical humanities, I do not lay claim to a new critical territory. Nor do I seek to promote a prescriptive methodology. Instead, my aim is to rationalise and consolidate diffuse tendencies along these lines that are already in practice. As an analytical framework for approaching the intersections of cultural, social, and economic formations of health, disease and the human body, I suggest a working definition of medicocultural materialism that is anchored in three primary characteristics:

- The first of these is an interest in diverse sources at the interface of the ‘medical’ and ‘cultural’, and a critical practice which insists on the conjunction, and parity, of the two. The expansive pool of primary materials from which such a practice draws may include (but is not limited to): scientific and medical journal articles and lectures, health advice literature, self-help manuals, clinical case notes, newspaper reports on scientific or medical topics, literary fiction, medical advertising, film and television. Working with these materials across disciplinary boundaries, the medicocultural materialist examines the ways in which ideas and concepts surrounding human health and the body are cocreated by both specialists and non-specialists.
- Second, medicocultural materialism inherits cultural materialism's combination of historicist analysis with scrutiny of our present condition. It therefore addresses the development of medical knowledge over time without shading into an ahistorical universalism which neglects to address key linguistic, conceptual and ideological changes.^iii^
- Third, in undertaking critical work within the medical humanities that is actively political, medicocultural materialism looks to the analyses of political economy within the discipline of medical sociology. The political economic critique focuses on the social and economic disadvantages of marginalised groups—women, non-whites, the aged, the unemployed and members of the working-class—and scrutinises scientific values and medical practices from a structuralist perspective that acknowledges their entanglement with the directives of capitalist productivism.^iv^

These key characteristics notwithstanding, any work seeking to bring cultural materialism into the orbit of the medical humanities must also address its theoretical and practical limitations. For example, while Williams' scholarship was highly sensitive to issues of class, it was less generous in its confrontations with questions surrounding gender and race (as Morag Shiach and Edward Said have noted, respectively).^v^ Scholarship drawing on this legacy must therefore acknowledge cultural materialism's own concessions to hegemonic ideologies, and work towards intersectionality.

In the sections that follow, I employ the medicocultural materialist approach outlined here to investigate the topic of blood rejuvenation. In exploring the ways in which blood's putative capacity to restore youth has been reported, represented and (re)imagined at the discursive intersections of science and culture, I draw on Williams' conceptualisation of ‘dominant, residual and emergent’ elements and Sinfield's notion of ‘faultlines’ to scrutinise the contradictions that emerge within their material formations.

## Blood and rejuvenation in 19th-century fin-de-siècle fiction

Associations between blood and youth did not emerge in the late 19th century, but can be traced back to antiquity.^vi^ An initial correlation between sanguinity and youth in humoral theory was sustained and developed by both medical speculation and mythological narratives throughout the mediaeval and early modern periods.^vii^ The first successful transfusion of blood from one human organism to another in 1829 further strengthened a prevailing conception of blood as substance with the power to reanimate the failing body.^viii^ However, in the closing decades of the 19th century, the notion that the ageing body could be restored by the infusion of young blood was boosted by further scientific developments and the emergence of new cultural forms. Longstanding interest in the regenerative potential of the blood was challenged in a new discourse that sought to identify it as the seat of corporeal degeneration. Physicians and social reformers focused on blood as a potential threat to health, and it was seen as the carrier of diseases including tuberculosis, cholera and syphilis and the material locus of inherited family traits such as feeble-mindedness and drunkenness (p. 262).^26^ But these new associations with impurity and degeneration did not dislodge older notions of blood as a regenerative substance with the capacity to revitalise ageing bodies. ‘Dominant’ associations of blood with disease and decline combined with ‘residual’ connotations of life, vitality and renewal.

The power of these residual connotations was such that when blood transfusion became a topic of intensive medical debate and discussion in fin-de-siècle medical literature, the procedure was readily configured as medical innovation, multivalent signifier and creative animus. In the journalism and fiction of an expanding popular print culture, dramatic tales of decrepit bodies reinvigorated by the procedure offered powerful narratives that newspaper editors on both sides of the Atlantic used to boost the circulation of their publications (refs. 27–30 p. 51). The pages of new mass-market magazines and periodicals, and the growth of circulating libraries and cheap paperbacks, offered fiction writers new opportunities for developing serial narratives and short stories for a broad readership. Blood rejuvenation became a recurring trope in new genres of UK and US popular fiction, as authors drew on renewed interest in blood transfusions and in the emerging social, political and cultural discourses surrounding old age. This fictional subgenre of blood rejuvenation—a thematic cognate of the ‘medical gothic’^ix^—was a material space in which writers negotiated the interplay of residual ideas about blood as a spiritual, mystical substance and its increasingly dominant meanings as biological material and economic commodity. In the faultlines of these texts, the structural social vulnerabilities of blood donors and the pecuniary value of blood as a covetable organic resource come to the surface.

In 1887, the American literary journal *The Argonaut* published a short story documenting ‘the startling results brought about by the transfusion of blood’.^31^ ‘The Man Who Grew Young Again’, by the Scottish-born, San-Francisco-based writer Robert Duncan Milne, follows the fate of a middle-aged English gentleman named Mr Wycherley, who sustains a gun-shot wound to his leg on a rural shooting expedition. The physician narrating the story suggests that the risks posed by blood loss are particularly high given Wycherley's advanced age—at 45, the injured man ‘does not possess the recuperative powers of a man 15 years his junior’ (p. 4). The blood transfusion the doctor subsequently performs represents a fusion of realism and fantasy, in which medical and cultural conceptualisations of blood intersect with dominant discourses on ageing masculinity. The doctor seeks ‘the youngest and richest human blood procurable’ to replace Wycherley's lost fluid. He selects two local brothers, ‘fine, strong, clear-skinned, strapping lads of some 20 summers … evidently of Teutonic origin’, on the basis that they possess ‘a practically unlimited store’ of ‘pure life-fluid’ (p. 4). The doctor improvises an experimental emergency transfusion, using blood vessels harvested from a recently slaughtered calf to prevent coagulation. Employing the services of both donors simultaneously, he positions the young men at either side of his patient and bridges the dying man's veins with each of theirs, fashioning a hybrid organism in which the blood of the old man and the younger men commingles. ‘A complete and perfect connection had now been formed with the circulatory organs of the men’, the narrator observes, so that ‘they formed, in fact, one circulatory system’ (p. 5). The brothers receive an initial $200 for their participation and a subsequent payment of $20 apiece for every day that they continue to supply the older man with their young blood.

Milne—who was educated at the University of Oxford and had a background in technology and science—envisages a parabiotic pairing of human subjects that recalls the experiments with animals pioneered by the French physiologist Paul Bert in the 1860s.^x^ But whereas Bert developed the technique as part of his investigations into organ transplantation, Milne envisages the regenerative effect of the introduction of young blood upon the aged organism. Over a period of days, Milne's fictional doctor begins to observe changes in the physical appearance and temperament of the older man and the young donors. United within this ‘curious physical trinity’, the youths begin to show signs of senescence, while the older man displays those of juvenescence (p. 5).^31^ ‘Structurally and organically affected by the new blood which was now circulating through his system’, the doctor observes, ‘Wycherley was indeed growing younger while his companions were growing proportionally older’ (p. 5). Sought initially as an emergency treatment for haemorrhage, the blood transfusion performed has a permanent rejuvenatory effect, conferring on the older man the appearance and sexual energy of youth. Restored to good health and to the physiological age and appearance of a person aged 28 years, Wycherley marries his son's young widow.

The explanation for Wycherley's transformation offered in Milne's story combines a humoral conceptualisation of blood as a sanguine element abundant in youth but decrescent over time, with a mechanistic understanding of its role in ‘the valvular action of the heart and the circulatory system’ (p. 4). Buried in the pages of literary magazine archives, Milne's tale has not received critical attention. Yet, the story testifies to a practical and creative interest in the possibilities of medically regenerating the ageing human body at the fin de siècle. Furthermore, the narrative's conventional resolution (medical recovery, marital union) cannot contain the disruptive power of its gothic elements. These reveal that the fantasy of rehabilitated senile male sexuality is predicated on the subjugation of the social and racial ‘other’. The two young ‘Teutonic’ farmhands, left ‘prematurely matured’ in the aftermath of their blood donations, attract no pity in the local community because it is well-known that they have been ‘paid liberally’ by the physician for ‘the loss of their vitality’ (p. 5). In this experimental medical marketplace, corporeal vitality—for which blood is the material conduit—is a commodity obtained by the socially dominant group at the expense of a less socially powerful one.

In the same year that Milne's tale appeared in the USA, another story featuring the rejuvenation of an old man through the transfusion of a younger man's blood was published in London, in a volume of short stories by the Irish popular fiction writer Richard Dowling. Like Milne's story, Dowling's tale, titled ‘Blood is Thicker than Water’, bears the same hallmarks of structural social inequality in its creative interpretation of blood's rejuvenatory capacity. In this instance, the old man is the Bishop of Barminster—a clergyman aged 70 years whose health is deteriorating. Assessing the bishop's condition, his unorthodox physician conceptualises ageing as a process that can be nullified by the nutritive and humoral value of young blood. The doctor settles on ‘a thick-set, bull-necked, ill-looking young man’ of disrepute who is nonetheless in ‘the prime of manhood…whose blood is dense with red corpuscles’ (pp. 12, 10).^33^ Following the procedure, the bishop makes a full recovery, and regains the athletic fervour of his youth. As an unsought consequence however, he also takes on aspects of the younger man's temperament, giving up his ministry to pursue new interests in boxing and betting. Dowling's tale draws on residual meanings of blood as the locus of human temperament and the source of bodily vitality, and probes these in relation to questions surrounding corporeal integrity and selfhood posed by the medical practice of moving blood between bodies. Ultimately however, the story's bromidic polarisation of esteemed, devotional cleric, on the one hand, and uneducated miscreant, on the other, does not traverse class distinctions, but reifies them instead.

In addition to Milne and Dowling, more widely discussed literary figures also drew inspiration from blood rejuvenation in the closing years of the 19th century. In 1896, the bestselling English popular novelist Mary Elizabeth Braddon published the short story ‘Good Lady Ducayne’ in an issue of *The Strand*, Britain's foremost general interest monthly magazine.^xi^ 34 The story's protagonist, Bella Rolleston, is a young girl aged 18 years from a modest background who becomes a hired companion to the aristocratic Lady Ducayne. On a trip to the Italian Riviera, Bella's health begins to deteriorate; once ‘ruddy and robust’, she grows increasingly ‘white and weak’ (p. 196). Noticing these changes, a young doctor of Bella's acquaintance named Herbert Stafford meets with Lady Ducayne, and the older woman presses him on his knowledge of ‘progressive science’ in the prolongation of human life (p. 197). Stafford deduces from lancet marks on Bella's arms that Lady Ducayne's private doctor has been draining the young girl's blood under sedation and transferring the substance to the older woman as an ‘experimental surgery’ in rejuvenation (p. 198). Stafford discovers that Lady Ducayne is in fact aged over 100 years, her unnatural lifespan facilitated by infusions of blood from two former young female companions who have not survived their exsanguination. With her ruse exposed, Lady Ducayne bestows a parting gift of a thousand pounds to Bella, together with instructions to invest the money in debenture stock, commanding the young woman to marry the young doctor who saved her.

In crafting this fiction, Braddon drew on folklore surrounding Elizabeth Bathory, the 16th-century Hungarian serial killer whose putative attempts to maintain her youth by bathing in the blood of the young women she killed earned her the popular title ‘the Blood Countess of Cathtice’.^36^ Framing the medical transformation of the female body at both ends of the life-course as a commercial practice, Braddon's tale mediates the dominant ideologies of class and gender at the fin de siècle. Bella, whose father abandoned her as a child, seeks a wage she can use to support both herself and her mother. Although she lacks pecuniary advantage, she possesses abundant physical capital—she is ‘fresh, blooming, a living image of youth and hope’ (p. 187).^34^ Lady Ducayne's deficiency is corporeal, rooted in her advanced years—she has ‘a withered old face under a plumed bonnet…so wasted by age that it seemed only a pair of eyes and a peaked chin’ (p. 187). This liability however, is offset by her abundant inherited wealth—she has paid her private doctor ‘thousands’ of pounds to boost her longevity by whatever means possible. Lady Ducayne's investment in Bella is founded on her belief that healthful vitality has its seat in the blood supply and that this constitution can be materially transmitted from one body to another. Her continuing health and prosperity are contingent on the exploitation of non-consenting, socially disadvantaged young women.

In the character of Lady Ducayne, dominant ideologies of gender and age intersect. This narrative portrait of an older woman's desperate desire for rejuvenation was published within a sociocultural landscape that was increasingly hostile towards the spectacle of female old age and yet squeamish about the mature woman's vanity.^37–40^ While Robert Duncan Milne envisaged the rejuvenation of his elderly male protagonist as an unexpected clinical outcome of transfusion-turned-parabiosis Braddon's Lady Ducayne is an appetitive consumer of blood-as-commodity. Milne's blood rejuvenation fantasy leaves his resexualised protagonist to live out his condition of renewed youth. Contrastively, the only seemly ending possible for the story told by ‘Good Lady Ducayne’ is one in which the rejuvenatory aspirations of the elderly woman yield to the superior value of authentic youth.

In the year following the publication of Braddon's story, Bram Stoker would explore the same themes of corporeal exploitation, intergenerational anxiety and blood rejuvenation in the fully realised trope of vampirism. *Dracula* has generated extraordinary critical interest since its publication in May 1897, its hermeneutic plurality augmented by a remarkable textual afterlife in popular culture.^41^ The modest body of scholarship that has attended specifically to the topic of blood in the novel has focused predominantly on its metaphorical and symbolic dimensions, and more recently, on its status as ‘scientific matter’^xii^.^4^ ^42^ One medicocultural materialist reading of blood rejuvenation in Stoker's novel might consider both the metaphorical and scientific meanings of blood, together with the power dynamics that inform its transference between bodies, and analyse both in relation to contemporary discourses of ageing.

Stoker's text adroitly navigates the epistemological plurality of the sanguine fluid. The coalescence of its residual meanings with new medical discourses and technologies is manifest in the descriptions of the four blood transfusions administered to Lucy Westenra throughout the course of the novel. But these transfusions yield a limited regenerative outcome only, temporarily restoring Lucy's youthful sensuality, but failing, ultimately, to save her life. Instead, true blood rejuvenation is achieved through the entirely supernatural and altogether more deviant act of blood-sucking.

The sexualised nature of Dracula's exploitation of young women through blood-taking is well-documented. So, too, is the notion that Stoker's vampire is a metaphor of capitalist consumption.^xiii^ Less attention, however, has been paid to the text's configurations of ageing and rejuvenation. Dracula's acts of sanguine consumption prolong his unnaturally long life and impart to him the physiological vitality of youth. Jonathan Harker's first encounter with the Count emphasises Dracula's age: he is ‘a tall old man, clean shaven save for a long white moustache’ (p. 22).^41^ Later in the novel, Harker discovers Dracula in his coffin, tumescent with Lucy's virginal blood:

looking as if his youth had been half renewed, for the white hair and moustache were changed to dark iron-grey; the cheeks were fuller, and the white skin seemed ruby-red underneath; the mouth was redder than ever, for on the lips were gouts of fresh blood, which trickled from the corners of the mouth and ran over the chin and neck … It seemed as if the whole awful creature were simply gorged with blood; he lay like a filthy leech, exhausted with his repletion (p. 59).

Historians of ageing have noted a proportional increase in the percentage of aged men and women in both the British and American populations from the early 19th century onwards.^44^ ^45^ Cross-disciplinary scholarship in history, sociology and political science has documented the ways in which ageing was reconstituted during this period as both a series of physiological changes and a socially constructed category of existence.^46–50^ The literary critic Karen Chase has suggested that ‘the Vampiric Complex reflects the fear among the young that the old are rapacious in their determination to cling to life even though the extension of their lives strips youth of their own’ (p. 134).^51^ We might thus read Dracula's habits of rejuvenatory exsanguination as the haematological cognate of his capitalist expansionism. From this perspective, the acts of blood rejuvenation in Stoker's novel can be understood as textual inscriptions of contemporary anxieties about both economic and generational monopolism.

Tracing the contradictions and irresolutions within these historical cultural texts discloses the features of disciplinary institutional practices as they pertain to the sanguine fluid. Possessed by bodies, transferrable and exploitable, in these literary fictions of transfusion and vampirism, blood is a vector for intersecting discourses of class, age, gender and medicine, and a signal for the appropriation and commodification of the human body. A recurrent feature of blood rejuvenation in these cultural forms is its dependence on the blood supply of disposable human subjects whose consent is either unsought, or circumscribed by their socioeconomic vulnerabilities.

## Present-day perspectives on blood rejuvenation

The practice of cultural materialism draws on historical perspectives to inform contemporary debates. With a definition of culture as active practice, the interplay of dominant, residual and emergent formations in a given society is not just past phenomenon, but continuing reality. How, then, might the analysis of fin-de-siècle medical gothic fictions of blood rejuvenation conducted above draw attention to the particular silences, tensions and contradictions in the present-day meanings and practices of blood rejuvenation?

Since the late 19th century, the meanings of blood have been further contested and transformed by a series of scientific developments (the discovery of blood groups in 1909, advancements in transfusion practices made during two world wars) and a number of public health crises (the proliferation of bloodborne diseases including syphilis, hepatitis and malaria, the biotechnologisation of blood extraction and transfusion, the AIDS epidemic).^2^ Yet, the conceptualisation of blood as a biological material with the capacity to transform the ageing body—and the structural social inequalities that often characterise practices of blood rejuvenation—persist across both the cultural imaginary and the sphere of medical research.

Over a century after Robert Duncan Milne envisaged the rejuvenatory outcome of the parabiotic pairing of young and old human subjects in ‘The Man Who Grew Young Again’, and decades after a group of biologists and gerontologists at Cornell University revived the technique first envisaged by Paul Bert in the 1860s,^xiv^ scientists at American universities are revisiting parabiosis as part of their investigations into the ageing of somatic stem cells. In one experiment, a mouse aged 2 months was surgically attached to an animal aged 23 months with cardiac hypertrophy. Four weeks of exposure to the circulatory systems of young mice had an antihypertrophic effect on the hearts of older mice, a change attributed to the proliferation of the protein GDF11, present in higher quantities in the blood of the young mice.^55^ Speaking to the *New Scientist*, Amy Wagers, Professor of Stem Cell and Regenerative Biology at Harvard University, noted that since all parts of the body age at the same rate, it is conceivable that the blood has an instrumental role in the ageing process.^56^

The scientific interest of other recent experiments in this area lies in neurodegeneration: the brain's diminished ability to produce new neurons, leading to impaired cognitive function. Hypothesising that blood has an instrumental role in these processes given its crucial function in supplying materials and oxygen to the brain, researchers reported that the stem cells of older mice exposed to youthful influences through heterochronic parabiosis acquired youthful potential, while those of the young mice lost their regenerative potential.^57–59^ Older mice that receive young blood show signs of regenerated muscle tissue, liver cell growth and improvements in synaptic plasticity. Meanwhile, young mice that receive blood from their older counterparts have demonstrated the structural and functional hallmarks of neural decline.^60^ In sum, the findings indicate that the introduction of young blood into old mice can modify cellular degeneration.

New research into blood rejuvenation has the potential to address human health conditions including anaemia, obesity, diabetes, high cholesterol and cancer. It also has significant implications for the treatment of diseases and disorders associated with ageing. Scientists at Stanford University are currently studying the effects of the introduction of young plasma to older people with Alzheimer's disease,^61^ while a clinical trial in South Korea is investigating whether human umbilical cord blood and plasma can favourably influence the ageing process.^62^ A medicocultural materialist approach to these developments insists on analyses of the sociocultural climate within which such practices—and their underlying rationales—are formulated. It also signals the considerable influence of economic factors in these developments, and their corresponding ethical dimensions.

Journalistic reports on the outcomes of the studies cited above are revealing of the ways in which the science of blood rejuvenation is still envisaged, discussed and represented in conjunction with its cultural associations and meanings. Cultural mediations of medical and scientific research in this area continue to generate ideological faultlines. In the UK, *The Independent*, *The Telegraph* and the *Metro* newspapers led their coverage of the 2014 findings on the rejuvenation of mice with headline references to ‘vampires’ or ‘vampire therapy’.^63–65^ Other reports on research into blood rejuvenation have invoked the ‘fountain of youth’.^66^ ^67^ Drawing on scholarship in science fiction studies that has emphasised the contributions of literary and filmic media to the ‘technoscientific imaginary’,^68^ ^69^ we might untangle the dominant, residual and emergent elements within these references. The invocation of vampirism in scientific reports on blood rejuvenation research, for example, conceivably evokes both anxiety and possibility. The figure of the vampire continues to elicit a strong fascination within popular culture. Over half of all vampire novels ever written were published in the first decade of the 21st century (p. 109).^70^ Aimed primarily at a teenage market, novel series within the popular subgenre of vampire romance such as *The Vampire Chronicles*^71^ and *Twilight*^72^ have exploited and reinforced the historical associations between blood rejuvenation and youthful sexuality. On the other hand, films such as *Thirst*,^73^ *Daybreakers*^74^ and *Only Lovers Left Alive*^75^ have explored the biopharmaceutical commodification of blood in dystopian fictions of vampirism.

The vampire is not a homogeneous signifier.^xv^ The notion of ‘vampire therapy’ may, for some, connote unnatural intervention or parasitic exploitation. For others, it may invite the thrill of the posthuman: a potential opportunity to transcend current corporeal limitations. Analyses of the cultural discourses of science journalism and literary fiction are crucial to critical perspectives on the public understanding of medical science. It is at this level of knowledge-making that expectations surrounding new developments in health and disease—and the agencies of both patient and patient-consumer—are formed. The imaginative possibilities generated by medical ideas and technologies at their early stages of development both emerge from, and contribute to, a general climate of uncertainty and speculation. Within this discursive environment, commercial opportunism can thrive.

A medicocultural materialist approach to blood rejuvenation that seeks continuities and disparities across disciplinary boundaries, that draws lines between past and present and that is ideologically alert, reveals instructive parallels. Take, for example, the correlation between the fictional Lady Ducayne's vampiric consumerism in fin-de-siècle commodity culture and blood rejuvenation treatments available in the contemporary medical marketplace. The second decade of the 21st century has seen the growth of the UK and the US markets for the platelet-rich fibrin matrix method (PRFM), a cosmetic surgical procedure in which platelets are extracted from a sample of a patient's blood and used as dermal fillers for the face. PRFM—more commonly referred to as the ‘vampire facial’—relies on the same conceptual model of blood as the one in Braddon's story, combining humoral and mythical associations with the putative authority of medical innovation in its appeal to the ageing woman. The success of the vampire facial, marketed primarily to female consumers and costing up to £2000, is contingent on the wealthy woman's readiness to believe that blood has the power to recondition the human form.^77–79^ Whether or not PRFM can live up to its rejuvenatory claims is an issue further complicated by the corporate agendas of its providers. The platelet extraction centrifuge method used to manufacture the serum was cleared by the US Food and Drug Administration (FDA) in 2002 for use by orthopaedic doctors to speed tissue repair. Platelets extracted in this way have not, however, been approved by the FDA for the purposes of facial rejuvenation, despite the claims made by PRFM supplier Selphyl in its promotional materials that it offers a ‘newly FDA-approved dermal filler’.^77^ A recent article in the *Archives of Facial Plastic Surgery* concluding that ‘PRFM treatment is a well-tolerated, excellent choice for use in the face’ was published by a paid consultant for the company Aesthetic Factors, which has developed the procedure.^80^

Similar concerns apply to other modern developments in blood rejuvenation. Bioethicists and journalists are drawing attention to the proliferation of for-profit clinical trials and unapproved stem cell clinics in the USA, including those that promote treatments for osteoarthritis and Alzheimer's disease.^81^ ^82^ Many of these studies—although advertised on Clinicaltrials.gov—do not require FDA approval.^xvi^ A clinical trial run by the California-based company Ambrosia to ‘evaluate the beneficial effects of infusions of plasma from young donors using blood biomarkers’, represents one such example.^83^ Willing participants aged 35 years or over must contribute $8000 in a pay-to-participate model recently passed by ethical review. Stanford neuroscientist Tony Wyss-Coray, who led the 2014 study in heterochronic parabiosis, has highlighted the absence of any clinical evidence that the treatment offered will be beneficial, citing Ambrosia's enterprise as an abuse of public trust in science and an exploitation of the general ‘excitement’ surrounding new anti-ageing solutions.^84^ There is much at stake here. In for-profit clinical trials, money is made prior to the drug being found safe and effective, and stem cell manipulation carries significant risks, including cancer.^67^ Even if the research outcome is favourable, a reliance on blood donations from young volunteers could lead to the growth of a black market in blood as a rejuvenatory commodity.^xvii^ 85

In their book *Tissue Economies: Blood, Organs, and Cell Lines in Late Capitalism*, Catherine Waldby (a medical sociologist) and Robert Mitchell (a professor of English), have problematised the ‘gift/commodity’ dichotomy, which has provided the dominant theoretical framework for understanding networks of tissue exchange in the global biomedical marketplace.^86^ Waldby and Mitchell argue that new forms of finance and venture capital in the realms of biotechnology and pharmaceuticals raise social and ethical concerns about the donation or sale of bodily tissue, the nature of informed consent and the beneficiaries of these transactions that the ‘gift/commodity’ model cannot accommodate. They call for renewed attention to how ‘the transfer of tissues from one person to another’ often follows ‘the trajectories of power and wealth’ (p. 8).

Meanwhile, public expectation surrounding anti-ageing innovation is high. Centuries-old mythical narratives about the fountain of youth and the elixir of life have been reinvigorated by experimental scientific techniques of blood rejuvenation, which offer the prospect of fantasy-turned-reality. The agencies of patients, consumers and participants are governed, at least in part, by imagination, and by dominant sociocultural standards of ‘ageing well’ that are expressive of neoliberal agendas. David Harvey has defined neoliberalism as ‘a theory of political economic practices that proposes that human well-being can best be advanced by liberating individual entrepreneurial freedoms and skills within an institutional framework characterised by strong private property rights, free markets and free trade’ (p. 2).^87^ The ‘emphatic turn’ towards neoliberalism in the global economy since the 1980s has resulted in policies of deregulation, austerity, privatisation and the erosion of welfare state provisions, including healthcare provisions (pp. 2–3). Yet, neoliberalism is more than a programme of economic liberalisation. It also operates as a hegemonic mode of discourse, ‘incorporated into the common-sense way many of us interpret, live in and understand the world’ (p. 3).

Reading contemporary research on parabiosis in relation to an understanding of neoliberalism as both an economic and a cultural logic draws attention not just to the risks of a deregulated market in private clinical trials, but to the capitalist appeal of ‘a regenerative body, whose every loss can be repaired’ (p. 30).^86^ Likewise, the market for vampire facials bears the hallmarks of a neoliberal emphasis on individual responsibility: the appeal to invest in one's own biological capital through cosmetic modification. Here, the trope of vampirism comes to signify the act of auto-exploitation—a self-commodifying behaviour incentivised by the dominant, ideologically charged rhetoric of ‘self-care’ and governed by the idea of the entrepreneurial self. Given the insidious character of claims on the human body such as these, an ideologically conscious hermeneutics of blood must be a crucial component in our continuing exploration of the substance's regenerative function and rejuvenatory potential.

## Conclusion: beyond blood

What might be gained from the application of a cultural materialist methodology to other themes and topics within the medical humanities? The case of blood rejuvenation suggests that an approach that is at once interdisciplinary, culturally oriented, historicist and comparative, can facilitate a more comprehensive and politically engaged understanding of the social, cultural and economic factors that influence processes of knowledge-making in relation to the medical sciences. In turn, the practice of cultural materialism within the medical humanities may have the potential to reinvigorate the tradition of Marxist critical theory across literary studies. The creeping extension of the neoliberal consensus within the academy has worked to stymy the growth of rigorous and innovative critique along these lines. There is an urgent need for a conscious, unified effort by scholars working across the medical humanities to push back against this trend by recovering the political dimensions of textual forms and the relations of domination and subordination they perpetually (re)inscribe.

In particular, medicocultural materialists might join with colleagues in medical sociology in recognising the primary characteristics of ‘medical neoliberalism’ and employing a range of critical tools to elucidate their specific instantiations. The medical sociologist Jill A. Fisher has drawn attention to the ways that medical neoliberalism prioritises ‘choice over equity and access’, ‘transforms patients into consumers’ and disaggregates the body, recommending its isolated parts for modification and enhancement (p. 65).^88^ Fisher notes that since the ruling by the FDA in 1997 permitting pharmaceutical companies to market their services to patients via direct-to-consumer advertising, the mass media has become the primary means by which participants are recruited for clinical trials in the USA (p. 65). American patients' perceptions of their health are therefore informed by powerful cultural narratives and concepts and by ‘targeted messages from the manufacturers of healthcare products’ (p. 66). Faced with such a system, a central objective of cultural materialist practice within the medical humanities must be to reconcile the appreciable benefits of scientific knowledge and experimentation with a searching critique of how the hegemonic influences that inform biomedical concepts and categories operate in the discursive spaces of everyday life.

## Footnotes

i Williams first used the term in a short essay published in the 100th issue of the journal *New Left Review* in 1976 and developed the approach more fully in his later works.^10^

ii The exceptions are reappraisals in the edited collection by Prendergast^14^ and in Milner,^15^ published in 1995 and 2002, respectively.

iii In his review of Anna Schaffner's *Exhaustion: A History*,^17^ Steffan Blayney poses the important ‘ontological question’: ‘How can we be sure, in comparing or concatenating concepts and diagnoses separated by time, place and language, that we are really writing the history of a single thing or idea? Once we decide on our object, we run the risk of finding it wherever we choose to look, imposing present-day concepts onto a period in which they are unfamiliar or inappropriate.^18^

iv For an overview of the political economy perspective in the sociology of health and illness, see Lupton (pp. 8–11).^19^

v Shiach points out that ‘Feminists can find much of use to them in the work of Raymond Williams; they cannot, however, find many women’ (p. 51).^20^ Edward Said observed Williams’ ‘relative neglect of the affiliation between imperialism and English culture’ (p. 314).^21^

vi Egyptian kings were said to have bathed in blood to heal sickness and to counter the infirmity of old age (p. 3).^22^

vii Galen mapped the four humours onto the four elements. A person predisposed to produce an excess of blood was of the sanguine temperament, typically characterised by warmth, enthusiasm and joy. The stage of life in which the sanguine temperament predominated was said to be youth.^23^ In 1492, an apocryphal story about Pope Innocent VIII receiving the blood of three young boys on his deathbed through veno-venous anastomosis circulated widely (p. 271).^24^

viii London physiologist James Blundell published an account of a transfusion he had successfully performed on a female patient suffering from postpartum haemorrhage. A number of physicians subsequently adopted the procedure as a treatment for blood loss.^25^

ix The category of the ‘medical gothic’ explores the intersections between Gothic studies and the critical medical humanities.^16^

x Bert was awarded the Prize of Experimental Physiology of the French Academy of Science in 1866 for this work, but his approach was not taken up again until the early 20th century, when Alexis Carrell developed a method of connecting blood vessels as part of his enquiry into organ ageing and the prolongation of life.^32^

xi Founded in 1891, *The Strand Magazine* was one of a group of general-interest family magazines which, at sixpence, made it half the cost of other magazines and affordable to a wider demographic including lower-middle-class and working-class readers.^35^

xii Willis notes Stoker's likely exposure to the discourses and practices of medicine, given that two of his brothers and his uncle were eminent physicians (pp. 303–304).^42^

xiii In analysing Stoker's novel, Franco Moretti, among others, has pointed to Marx's configuration of capital as ‘dead labour which, vampire-like, lives only by sucking living labour, and lives the more, the more labour it sucks’ (p. 91).^43^

xiv Joining rats of different ages, the Cornell team provided evidence that the older parabiont displayed signs of improved longevity and tissue function.^52–54^ This was followed by subsequent research in the 1970s into the effect of heterochronic parabiosis on the lifespan, and Russian use of the technique in the last two decades of the 20th century to study the physiology of ageing (p. 526).^32^

xv For analyses of the ways in which the figure of the vampire has been consistently reinterpreted in relation to the shifting social, cultural, and political contexts of the twentieth century, see Auerbach.^76^

xvi Bazar^82^ notes that Clinicaltrials.gov does not vet its listings or provide details of associated costs for participants.

xvii The National Organ Transplant Act, which outlaws the sale of soft tissue organs, excludes plasma. In the USA, donors can earn up to $200 a month for plasma donations.^85^

## References

[1] Bradburne JM Perspectives on art, power, politics and pathology. In: Bradburne JM Blood: art, power, politics, and pathology. Prestel, 2001:11–20.

[2] Starr D Blood: an epic history of medicine and commerce. Little, Brown and Company: 1999.

[3] Pelis K Blood standards and failed fluids: clinic, lab, and transfusion solutions in London, 1868–1916. Hist Sci 2001;39:185–213. http://adsabs.harvard.edu/full/2001HisSc..39..185P

[4] Stephanou A A “ghastly operation”: transfusing blood, science and the supernatural in vampire texts. Gothic Stud 2013 15:53–65. 10.7227/GS.15.2.4

[5] rejuvenate, v. OED Online [Internet]. [cited 13 January 2017] Oxford University Press: 2017.

[6] rejuvenation, n. OED Online [Internet]. [cited 13 January 2017] Oxford University Press: 2017.

[7] Viney W, Callard F, Woods A Critical medical humanities: embracing entanglement, taking risks. Med Humanit 2015;41:2–7. 10.1136/medhum-2015–010692 26052111 PMC4484495

[8] Nelson C Cultural studies: an introduction. In: Grossberg L, Nelson C, Treichler PA Cultural studies. Routledge, 2002:1–16.

[9] Lewis BE Reading cultural studies of medicine. J Med Humanit 1998;19:9–24. 10.1023/A:1024931817150

[10] Williams R Notes on Marxism in Britain since 1945. New Left Review 1976;I/100:81–94. https://newleftreview.org/I/100/raymond-williams-notes-on-british-marxism-since-1945.

[11] Williams R Marxism and literature. Oxford University Press: 1977.

[12] Dollimore J, Sinfield A Political Shakespeare: essays in cultural materialism. Manchester University Press: 1994 [1985].

[13] Sinfield A Faultlines: cultural materialism and the politics of dissident reading. Clarendon Press: 1992.

[14] Prendergast C Cultural materialism. University of Minnesota Press: 1995.

[15] Milner A Re-imagining cultural studies: the promise of cultural materialism. Sage: 2002.

[16] Wasson S Useful darkness: intersections between medical humanities and gothic studies. Gothic Stud 2015;17:1–12. 10.7227/GS.17.1.1

[17] Schaffner AK Exhaustion: a history. Columbia University Press: 2016.

[18] Blayney S Book review: exhaustion: a history. In: Med Humanit. BMJ BLOGS. 9 August 2016 [cited 13 January 2017] http://blogs.bmj.com/medical-humanities/2016/08/09/book-review-exhaustion-a-history/

[19] Lupton D Medicine as culture: illness, disease and the body in western societies. Sage: 2003.

[20] Shiach M A gendered history of cultural categories. In Prendergast C Cultural materialism. University of Minnesota Press, 1995:51–70.

[21] Thompson EP, Said EW Last dispatches from the border country: Raymond Williams, 1921-1988. Nation 1988;246:310–14. The Nation Archive Premium Edition, Ipswich, MA.

[22] Learoyd P The history of blood transfusion prior to the 20th century: Part I. Transfus Med 2012;22:308–14. 10.1111/j.1365–3148.2012.01180.x. 22994447

[23] Galen. De temperamentis. In: Payne JR Galeni Pergamensis, De temperamentis, et de inaequali intemperie. C. J. Clayfor, A. Macmillan and R. Bowes, 1881 [cited 13 January 2017] https://archive.org/details/galenipergamens01payngoog

[24] Rivera AM, Strauss KW, Van Zundert A, et al The history of peripheral intravenous catheters: how little plastic tubes revolutionized medicine. Acta Anaesthesiol Belg 2005;56:271–82. http://www.ncbi.nlm.nih.gov/pubmed/16265830 16265830

[25] Blundell J Successful case of transfusion. Lancet 1829;1:431–2.

[26] Fröhlich S, Davidson M Introduction: blood bound. J Literary Cultur Disabil Stud 2016;10:261–9. 10.3828/jlcds.2016.23

[27] Lederer SE Flesh and blood: organ transplantation and blood transfusion in twentieth-century America. Oxford University Press: 2008, p. 51.

[28] Transfusion of blood. The Dundee Courier & Argus (Dundee, Scotland). 1882 Mon 25 Sept: 4. [cited 13 January 2017]. British Library Newspapers, Part II: 1800–1900 http://gale.cengage.co.uk/british-library-newspapers/19th-century-british-library-newspapers-part-ii.aspx

[29] The transfusion of blood. *The Evening News* (Portsmouth, England). 1883 Tues 13 Mar: 4. [cited 13 January 2017]. British Library Newspapers, Part III: 1741–1950 http://gale.cengage.co.uk/british-library-newspapers/british-newspapers-part-iii.aspx

[30] Science and invention. *The Newcastle Courant* (Newcastle-upon-Tyne, England). 1882 Fri 24 Nov: 6. [cited 13 January 2017]. British Library Newspapers, Part I: 1800–1900 http://gale.cengage.co.uk/british-library-newspapers/19th-century-british-library-newspapers-part-i.aspx

[31] Milne RD The man who grew young again. The Argonaut 1887;20:4–6.

[32] Conboy MJ, Conboy IM, Rando TA Heterochronic parabiosis: historical perspective and methodological considerations for studies of aging and longevity. Aging Cell 2013;12:525–30. 10.1111/acel.12065 23489470 PMC4072458

[33] Dowling R Blood is thicker than water. In: Dowling R With the unhanged. Swan Sonnenschein, Lowrey & Company, 1887:1–21.

[34] Braddon ME Good lady Ducayne. *The Strand Magazine* XI. 1896 Feb: pp. 185–99. [cited 13 January 2017] https://archive.org/stream/TheStrandMagazineAnIllustratedMonthly/TheStrandMagazine1896aVol.XiJan-jun#page/n0/mode/2up

[35] Ashley A The age of the storytellers: British popular fiction magazines 1880–1950. Oak Knoll Press, 2006:1, 11, 196–197.

[36] McNally RT Dracula was a woman: in search of the blood countess of Transylvania. McGraw-Hill: 1983.

[37] Heath K “How to keep young”: advertising and late-Victorian anxiety. In Heath K Aging by the book: the emergence of midlife in Victorian Britain. State University of New York Press, 2009:171–98.

[38] Niles L Malthusian menopause: aging and sexuality in Elizabeth Gaskell's *Cranford*. Vic Lit Cult 2005;33:293–310. 10.1017/S1060150305000859

[39] Niles L Owning “the dreadful truth”: or, is thirty-five too old? Age and the marriageable body in Wilkie Collins's Armadale. Nineteenth-Century Lit 2010;65:65–92. 10.1525/ncl.2010.65.1.65

[40] Small H The double standard of aging: on missing Stendhal in England. In: Boehm K, Farkas A, Zwierlein A Interdisciplinary perspectives on aging in nineteenth-century culture. Routledge, 2013:210–31.

[41] Stoker B Dracula. Penguin Classics: 2003 [1897].

[42] Willis M “The invisible giant”: *Dracula*, and disease. Stud Novel 2007;39:301–25. http://www.jstor.org/stable/29533817

[43] Moretti F Signs taken for wonders: essays in the sociology of literary forms. Verso: 1988.

[44] Johnson P Old age and ageing in Britain. Refresh [Internet] 17, 1993 [cited 13 January 2017]; Autumn: 5–8 http://www.ehs.org.uk/dotAsset/12fc863a-7dba-4787-9307-3ec5bb2f3b6d.pdf

[45] Achenbaum WA Old age in the New Land: the American experience since 1970. The John Hopkins University Press, 1978:58–66.

[46] Quadagno J Aging in early industrial society: work, family, and social policy in nineteenth-century England. Academic Press: 1982.

[47] Cole TR The journey of life: a cultural history of aging in America. Cambridge University Press: 1992.

[48] Haber C Beyond sixty-five: the dilemma of old age in America's Past. Cambridge University Press: 1985.

[49] Katz S Disciplining old age: the formation of gerontological knowledge. University of Virginia Press: 1996.

[50] Thane P Old Age in English history. Oxford University Press: 2000.

[51] Chase K “Senile” sexuality. In: Boehm K, Farkas A, Zwierlein A Interdisciplinary perspectives on aging in nineteenth-century culture. Routledge, 2013:132–46.

[52] Pope F, Lunsford W, McCay CM Experimental prolongation of the life span. J Chronic Dis 1956;4:153–8 10.1016/0021–9681(56)90015–7. 13345850

[53] McCay CM, Pope F, Lunsford W, et al Parabiosis between young and old rats. Gerontologia 1957;1:7–17 10.1159/000210677. 13405201

[54] Lunsford WR, McCay CM, Lupien PJ, et al Parabiosis as a method for studying factors which affect aging in rats. Gerontologia 1963;7:1–8. 10.1159/000211170 13931735

[55] Loffredo FS, Steinhauser ML, Jay SM, et al Growth differentiation factor 11 is a circulating factor that reverses age-related cardiac hypertrophy. Cell 2013;153:828–39. 10.1016/j.cell.2013.04.015 23663781 PMC3677132

[56] Heaven D Young blood reverses heart decline in old mice. In: New scientist. 9 May 2013 [cited 13 January 2017] http://www.newscientist.com/article/dn23511-young-blood-reverses-heart-decline-in-old-mice/

[57] Conboy IM, Conboy M, Wagers AJ, et al Rejuvenation of aged progenitor cells by exposure to a young systemic environment. Nature 2005;433:760–4. 10.1038/nature03260 15716955

[58] Villeda SA, Luo J, Mosher KI, et al The ageing systemic milieu negatively regulates neurogenesis and cognitive function. Nature 2011;477:90–4. 10.1038/nature10357 21886162 PMC3170097

[59] Villeda SA, Wyss-Coray T The circulatory systemic environment as a modulator of neurogenesis and brain aging. Autoimmun Rev 2013;12:674–7. 10.1016/j.autrev.2012.10.014 23201925

[60] Villeda SA, Plambeck KE, Middeldorp J, et al Young blood reverses age-related impairments in cognitive function and synaptic plasticity in mice. Nat Med 2014;20:659–63. 10.1038/nm.3569 24793238 PMC4224436

[61] Clinicaltrials.gov. [Internet]. The Plasma for Alzheimer Symptom Amelioration Study. [updated 2016 November; cited 13 January 2017] https://clinicaltrials.gov/ct2/show/NCT02256306

[62] Clinicaltrials.gov. [Internet]. Clinical Trial to Evaluate the Potential Efficacy and Safety of Human Umbilical Cord Blood and Plasma. [updated 2016 January; cited 13 January 2017] https://clinicaltrials.gov/ct2/show/NCT02418013

[63] Von Radowitz J Vampire therapy: young blood may reverse ageing. In: *Independent*. 4 May 2014 [cited 13 January 2017] http://www.independent.co.uk/news/science/vampire-therapy-young-blood-may-reverse-ageing-9323042.html

[64] Knapton S “Vampire therapy” could reverse ageing, scientists find. In: Telegraph. 4 May 2014 [cited 13 January 2017] http://www.telegraph.co.uk/news/science/science-news/10807478/Vampire-therapy-could-reverse-ageing-scientists-find.html

[65] McQueeney K Forever young: can “vampire therapy” blood injections really reverse the ageing process? In: Metro. 4 May 2014 http://metro.co.uk/2014/05/05/forever-young-can-vampire-therapy-blood-injections-really-reverse-the-ageing-process-4718661/

[66] Eck A Mouse experiments hint at fountain of youth in young blood. In: Nova Next. 5 May 2014 [cited 13 January 2017] http://www.pbs.org/wgbh/nova/next/body/young-blood-might-rejuvenate-older-adults/

[67] Krakowski A Young blood: chasing the fountain of youth. In: The Naked Scientists. University of Cambridge 8 October 2015 [cited 13 January 2017] http://www.thenakedscientists.com/HTML/articles/article/young-blood-chasing-the-fountain-of-youth/

[68] Miller G, McFarlane M Science fiction and the medical humanities. Med Humanit 2016;42:213–18. 10.1136/medhum-2016–011144 27885035

[69] Kirby DA Lab coats in Hollywood: science, scientists and cinema. MIT: 2011.

[70] Melton JG, Hornick A The vampire in folklore, history, literature, film and television: a complete bibliography. McFarland: 2015.

[71] Rice A The vampire chronicles [novel series]. Knopf: 1976–2016.

[72] Meyer M Twilight [novel series] Little, Brown and Company: 2005–2008.

[73] *Thirst*. [film] Moho Films/Focus Features International: 2009.

[74] *Daybreakers*. [film]. Lionsgate: 2009.

[75] *Only Lovers Left Alive*. [film]. Recorded Picture Company/Pandora Film: 2013.

[76] Auerbach N Our demons, ourselves. University of Chicago Press: 1995.

[77] Saint Louis C “Vampire facelifts”: smooth at first bite. 2 March 2011. [cited 13 January 2017]. In: *New York Times* http://www.nytimes.com/2011/03/03/fashion/WEBSkin.html

[78] Chalmers S Vampire facials: the cold facts. 7 July 2014 [cited 13 January 2017]. In: *Telegraph* http://www.telegraph.co.uk/news/health/10949952/Vampire-facials-the-cold-facts.html

[79] Rose D What is a vampire facial? 22 July 2014 [cited 13 January 2017]. In: *Telegraph* [Internet] http://www.telegraph.co.uk/beauty/skin/what-is-a-vampire-facial/

[80] Sclafani AP Safety, efficacy, and utility of platelet-rich fibrin matrix in facial plastic surgery. Arch Facial Plast Surg 2011;13:241–51. 10.1001/archfacial.2011.3 21339469

[81] McGinley L Unregulated stem-cell clinics are proliferating across the United States. 30 June 2016 [cited 13 January 2017]. In: *Washington Post* https://www.washingtonpost.com/news/to-your-health/wp/2016/06/30/unregulated-stem-cell-clinics-are-proliferating-across-the-u-s/?utm_term=.f11a135b7ab7

[82] Bazar E Want to enroll in a clinical trial? NIH database is huge—but lacks a few key details. 26 July 2016 [cited 13 January 2017]. In: *Washington Post* https://www.washingtonpost.com/national/health-science/want-to-enroll-in-a-clinical-trial-nih-database-is-huge--but-lacks-a-few-key-details/2016/07/26/52e5eda8-4518-11e6-88d0-6adee48be8bc_story.html?utm_term=.73e032128a92

[83] Clinicaltrials.gov. [Internet]. Young Donor Plasma Transfusion and Age-Related Biomarkers. [updated 2016 October; cited 13 January 2017] https://clinicaltrials.gov/show/NCT02803554

[84] Kaiser J Young blood antiaging trial raises questions. 1 August 2016 [cited 13 January 2017]. In: *Science* http://www.sciencemag.org/news/2016/08/young-blood-antiaging-trial-raises-questions

[85] Interlandi J What are the ethics of using young blood to reverse the effects of aging? 8 October 2015 [cited 13 January 2017]. *TED* http://ideas.ted.com/what-are-the-ethics-of-using-young-blood-to-reverse-the-effects-of-aging/

[86] Mitchell R, Waldby C Tissue economies: blood, organs and cell lines in late capitalism. Duke University Press: 2006.

[87] Harvey D A brief history of neoliberalism. Oxford University Press: 2005.

[88] Fisher JA Coming soon to a physician near you: medical neoliberalism and pharmaceutical clinical trials. Harvard Health Policy Rev 2007;8:61–70. http://www.hhpronline.org/past-print-issues/2007 21572938 PMC3092550

